# Predictors of histopathological esophagitis in infants and adolescents with esophageal atresia within a national follow-up programme

**DOI:** 10.1371/journal.pone.0266995

**Published:** 2022-04-15

**Authors:** Felipe Donoso, Anna Beckman, Andrei Malinovschi, Helene Engstrand Lilja

**Affiliations:** 1 Department of Women’s and Children’s Health, Uppsala University, Uppsala, Sweden; 2 Section of Pediatric Surgery, Uppsala University Children’s Hospital, Uppsala, Sweden; 3 Department of Medical Sciences, Clinical Physiology, Uppsala University, Uppsala, Sweden; Istanbul University Cerrahpasa Faculty of Medicine: Istanbul Universitesi-Cerrahpasa Cerrahpasa Tip Fakultesi, TURKEY

## Abstract

**Purpose:**

Esophageal atresia (EA) is a congenital anomaly of the foregut. Although the survival has improved over the years there is a significant gastrointestinal morbidity affecting physical function and health-related quality of life. The aims of the study were to identify and evaluate predictors of histopathological esophagitis in infants and adolescents with EA.

**Methods:**

Single centre, cross-sectional study including one and 15-year-old patients operated for EA that participated in the national follow-up programme between 2012 and 2020 according to a pre-established protocol including upper endoscopy with oesophageal biopsies and 24h-pH-test. Data was collected from patients’ medical records and pH-analysis software. Regression models were used to identify predictors of histopathological oesophagitis. Possible predictors were abnormal reflux index, endoscopic esophagitis, hiatal hernia, symptoms of gastroesophageal reflux (GER) and age.

**Results:**

65 patients were included, 47 children and 18 adolescents. All children were treated with PPI during their first year of life. Symptoms of GER were reported by 13 (31.7%) of the infant’s caregivers, 34 of the children (72.3%) had abnormal reflux index and 32 (68.1%) had histopathological esophagitis. The corresponding numbers for adolescents were 8 (50%), 15 (83.3%) and 10 (55.6%). We found no significant associations between histopathological esophagitis and endoscopic esophagitis, symptoms of GER, hiatus hernia or age group. Abnormal reflux index was an independent predictor of histopathological esophagitis. Seven patients with normal reflux index had histopathological esophagitis, all grade I.

**Conclusions:**

We found a high prevalence of histopathological esophagitis despite PPI treatment in accordance with recommendations. No significant difference between the two age groups was seen. Abnormal reflux index was an independent predictor of histopathological esophagitis. However, we cannot recommend the use of pH-metry as a substitute for esophageal biopsies; future studies are needed to elucidate if esophageal biopsies might be postponed in infants with normal reflux index.

## Introduction

Esophageal atresia (EA) is a congenital anomaly of the foregut affecting about 1:3000–4500 live births [1]. Although the prognosis for infants born with EA has improved over the years with survival rates approaching 90% [1], there is significant gastrointestinal and respiratory morbidity [2, 3] in survivors affecting physical function and health-related quality of life (HRQoL) [4–6]. Gastro-intestinal morbidity includes gastroesophageal reflux disease (GERD), esophagitis, eosinophilic esophagitis, dysphagia [7] and esophageal strictures [2]. Chronic gastroesophageal reflux (GER) might lead to mucosal damage, Barrett’s esophagus and occasionally esophageal cancer [8]. GER appears to be due to abnormal esophageal motility [9–12], dysmotility of the stomach [13], with slow gastric emptying [14]. Primary malformations of the nerve supply and intraoperative damage to the nerve supplies of the esophagus may be other mechanisms resulting in GER [15–17].

During the last decade several centres have developed follow-up programmes for patients treated for EA. These programmes often include evaluation of symptoms of GER, 24h-pH or pH-impedance monitoring and endoscopy of upper gastrointestinal tract as recommended by ESPGHAN/ NASPGHAN [18] and ERNICA [19]. One of the goals of EA follow-up programmes is to diagnose complications such as esophagitis, GERD and strictures. The timing of the endoscopic procedure is controversial and despite consensus documents the evidence level for the recommendations are low (ESPGHAN/ NASPGHAN [18] statement 7, similar in ERNICA [19] statement 18 and 19). A particularly problematic group, are the asymptomatic patients, where the proposed algorithm by ESPGHAN/ NASPGHAN [18] does not include endoscopy at discontinuation of PPI and they rely on the presence of symptoms of GER for recommending early endoscopy. On the other hand, endoscopic procedures in children requires anaesthesia which has raised concerns that are gaining more support given reports on anaesthesia’s effects on children’s neurodevelopment [20–24]. As mentioned earlier, the evidence in the topic is low and there are few studies in children and adolescents treated for EA that consider histopathological esophagitis as the outcome [25–29].

The aims of the study were to identify and evaluate predictors of histopathological esophagitis in infants and adolescents with EA.

## Methods

### Patients

**Fig 1:**
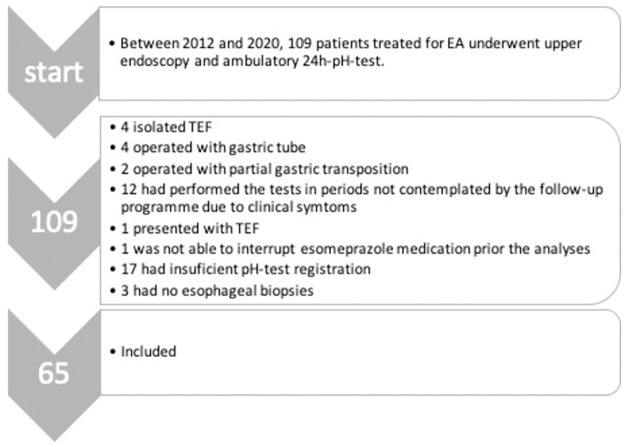
Flow chart of the patient inclusion process.

This was a cross-sectional study including patients who had undergone surgical correction of EA at University Children’s Hospital (Uppsala, Sweden) between January 1994 and June 2018 and who had participated in the national follow-up programme for one or 15-year-old patients between 2012–2020. The exclusion criteria were: surgical replacement therapy (gastric tube, gastric/intestinal transposition); patients with isolated tracheoesophageal fistula (TEF) (Gross type E); follow-up in period not stipulated by the follow-up protocol but initiated on clinical basis; ambulatory 24h-pH-test with a recording less than 20h; patients that discontinued esomeprazole treatment more than eight weeks prior to the diagnostic procedures; inability to discontinue esomeprazole treatment four weeks prior to the pH-test; lack of biopsy (Fig 1). The repair operations were mainly performed by three senior surgeons and the endoscopic procedures were performed by four experienced surgeons. The use of patient data in this study was approved by the Regional Ethical Review Board in Uppsala, Sweden (Dnr 2014/060, 2014/119/1 and 2014/1191/3). The ethics committee waived the requirements for informed consent. Data was not anonymised prior to the collection by the authors.

### National follow-up programme

A national follow-up programme after EA repair was established in 2011 in Sweden. The programme has a multidisciplinary approach with a team consisting of a paediatric surgeon, a paediatric pulmonologist and a dietitian. At one and 15 years of age the patients were evaluated by the members of the team through a structured interview. An upper endoscopy with the collection of biopsies from the esophagus was performed. The biopsies were fixed in formaldehyde, stained according to clinical protocols and evaluated by clinical pathologists. All children received PPI medication esomeprazole 1-2mg/kg/day after repair of EA until one year of age. PPI was discontinued four weeks prior to pH-metry. PPI treatment continued if histopathology showed esophagitis. A pH/impedance catheter (Versaflex^®^ Z, Sierra Scientific Instruments Inc., 5757 W Century Blvd Suite 660, Los Angeles, CA 90045 United States) was placed during the endoscopic procedure under visual control to ensure proper placement according to methodological requirements. The ambulatory 24h-pH-tests were conducted using the recording device Digitrapper^®^ pH-Z and AccuView^™^ analysis software (Medtronic, Operational Headquarters 710 Medtronic Parkway, Minneapolis, MN 55432–5604, USA). To monitor symptoms, body position, and intake of food and liquids, adolescents and caregivers kept a diary during pH-metry.

### Data collection and definitions

Data was collected from patients’ medical records and pH analysis software, regarding: basic patient characteristics, type of esophageal correction, complications, follow-up interviews and upper endoscopy protocols; ambulatory 24h-pH-test analysis protocols and clinical interpretation given by clinical physiologists; and biopsy evaluation as described above.

Despite the benefits of combined multiple intraluminal impedance (MII) and pH-metry (i.e., pH-impedance monitoring) over just pH-metry, only the pH data was analysed. The reason for this was that only automated analysis of impedance data was available and it has been shown by others [29–31] that automated analysis without subsequent manual correction leads to both over- and underestimation of the reflux burden in patients with EA.

Histopathological esophagitis was defined as esophagitis grade I-III according to Ismail-Beigi [32, 33] and computed as a dichotomous variable. The biopsies were also evaluated for intestinal metaplasia and dysplasia (presence of abnormal cells). Boix-Ochoa scoring system was used to evaluate GER. The score has six components; percentage time with acidic reflux (pH < 4) for the total period, percentage time with acidic reflux in the upright position, percentage time with acidic reflux in supine position, number of reflux episodes, number of reflux episodes of 5 min or more, longest reflux episode and percentage time with acidic reflux in the prone position [34]. Boix-Ochoa score above the 95^th^ percentile (>11.99) was considered to be abnormal reflux index and treated as a dichotomous variable. Endoscopic esophagitis was defined as signs of inflammation in the esophageal mucosa described by the surgeon (areas of erythema, erosions, coating, ulcers as in Savary-Miller classification) and was coded as a dichotomous variable. Hiatal hernia was defined as a gap at the level of the lower esophageal sphincter around the scope in the inverted gastric view. Patient/caregiver reports of regurgitation, vomiting and heartburn were considered symptoms of GER in patients that did not have an obvious alternative explanation such as an anastomotic stricture that required dilatation during upper endoscopy. Dysphagia was defined as reported aspiration during swallowing, difficulties swallowing specific food or consistency of food. Anastomotic stricture was defined as a symptomatic narrowing of the esophagus seen on upper endoscopy that required at least one dilatation. Abnormal diet for age was applied for patients that did not tolerate age-appropriate food consistency or who used gastrostomy. Weight and length of the patients at follow-up were measured in z-score according to the WHO and Fenton growth charts [35–38].

### Statistics

Possible predictors of histopathological esophagitis were identified from the literature and based on clinical use and statistical limitation of our sample size they were limited to five predictors; abnormal reflux index, endoscopic esophagitis, hiatal hernia, symptoms of GER and age group. These predictors were included in different logistic regression models; a simple logistic regression and a multiple logistic regression with stepwise forward selection based on Akaike’s Information Criteria (AIC) to evaluate improvement of the model. The multiple logistic regression included all five possible predictors mentioned above.

The study size was based on the inclusion of all patients that fulfilled the inclusion criteria and no formal sample size calculation was performed for the present study.

Statistical analyses were performed using R version 4.0.1 Copyright (C) 2020 The R Foundation for Statistical Computing with R Commander v 2.6–2 and the EZR v1.42 plug-in.

The anthropometric data was compared to the reference values provided by the WHO Child Growth Standards [37, 38] and the extension of this data published by Rodd et al. [35] using the online CPEG Shiny App “WHO Z-scores 0-19y” [39] for follow-up data and reference values for gestational age week 22–50 published by Fenton et al. [40] using the “preterm Z-score” app for birth data.

### Patient and public involvement statement

This study is based on data from the Swedish national follow-up programme, the patients/caregivers or the public were not involved in the development of the study.

## Results

Between 2012 and 2020, 109 patients treated for EA underwent upper endoscopy with esophageal biopsies and ambulatory 24h-pH-test. Out of the 109 patients, 65 were included in the study. The reasons for patient exclusion are given in Fig 1.

The demographics of the children and adolescents are summarised in Table 1. All children were treated with PPI during the first year of life. Due to the spread in age at the follow-up of children, 12 patients (25.5% of children) had discontinued PPI-treatment for over eight weeks before the control. Five patients in the adolescents’ group (31.2% of adolescents) were treated with PPI. As described in Table 2, at one-year follow-up, 72.3% of the children (n = 34) had abnormal reflux index and 68.1% (n = 32) had histopathological esophagitis. The corresponding numbers for the adolescents were 83.3% (n = 15) and 55.6% (n = 10). The proportion of children with endoscopic esophagitis was 21.3% (n = 10) and 27.8% (n = 5) of adolescents. Clinical symptoms of GER were reported by 31.7% (n = 13, NA = 6) of the children’s caregivers and by 50% (n = 8, NA = 2) of the adolescents. Seven patients had a gastrostomy, five of them had abnormal reflux index with Boix-Ochoa score ranging from 12 to 235.1. Three patients in the study group (4.6%) underwent Nissen fundoplication.

**Table 1:** Patient demographics by age group.

Factor	Group	Children (n = 47)	Adolescents (n = 18)
Age		1.19 [1.08, 1.51]	15.17 [14.61, 15.47]
Male sex (%)		25 (53.2)	9 (50.0)
Gestational age, week (median [IQR])		38.86 [36.57, 40.0]	38.0 [33.79, 39.54]
Birth length Z-score (median [IQR])		0.05 [-0.44, 0.60] NA = 1	0.57 [-1.04, 0.76]
Birth weight Z-score (median [IQR])		-0.44 [-0.93, 0.38]	-0.33 [-0.96, 0.27]
Gross type (%)	A	2 (4.3)	0 (0.0)
	B	1 (2.1)	0 (0.0)
	C	41 (87.2)	18 (100.0)
	D	3 (6.4)	0 (0.0)
	E	0 (0.0)	0 (0.0)
Type of esophageal correction* (%)	PDA	44 (93.6)	18 (100.0)
	DPA	3 (6.4)	0 (0.0)
Anastomotic stricture (%)		13 (27.7)	9 (50.0)
Nissen fundoplication (%)		1 (2.1)	2 (11.1)
**At follow up**			
Z-score for height (median [IQR])		0.02 [-1.42, 0.83] NA = 26	0.18 [-1.01, 0.59] NA = 3
Z-score for weight (median [IQR])		-0.35 [-1.20, 0.22] NA = 5	0.33 [-1.38, 0.87] NA = 2
Proton pump inhibitor^ (%)		35 (74.5)	5 (31.2) NA = 2
Symptoms of GER (%)		13 (31.7) NA = 6	8 (50.0) NA = 2
Dysphagia (%)		24 (52.2) NA = 1	5 (29.4) NA = 1
Abnormal diet for age (%)		20 (43.5) NA = 1	2 (11.8) NA = 1
Gastrostomy (%)		6 (12.8)	1 (5.6)

*PDA: primary direct anastomosis; DPA: delayed primary anastomosis.

^At follow-up, all children were treated at least until 1 year of age.

**Table 2:** Follow-up results by group.

Factor		Children (n = 47)	Adolescents (n = 18)	Esophagitis^+^ (n = 42)	No esophagitis (n = 23)
**Endoscopy**					
Esophagitis (%)		10 (21.3)	5 (27.8)	13 (31.0)	2 (8.7)
Hiatal hernia (%)		13 (30.2) NA = 4	1 (5.9) NA = 1	10 (27.0) NA = 5	4 (17.4)
**Histopathology**					
Esophagitis (%)		32 (68.1)	10 (55.6)	-	-
No esophagitis (%)		15 (31.9)	8 (44.4)	-	-
Inflammation grade^+^	I	21 (44.7)	7 (38.9)	28 (66.7)	0 (0.0)
	II	10 (21.3)	3 (16.7)	13 (31.0)	0 (0.0)
	III	1 (2.1)	0 (0.0)	1 (2.4)	0 (0.0)
Dysplasia (%)		0 (0.0)	0 (0.0)	0 (0.0)	0 (0.0)
Intestinal metaplasia (%)		1 (2.4)	0 (0.0)	1 (2.4)	0 (0.0)
**pH test**					
Boix-Ochoa median (IQR)		43.90	22.60	44.90	24.00
		[11.30, 61.65]	[17.45, 43.77]	[17.97, 63.35]	[9.80, 43.20]
Abnormal reflux index (%)		34 (72.3)	15 (83.3)	35 (83.3)	14 (60.9)

^+^According to Ismail-Beigi [32].

**Fig 2:**
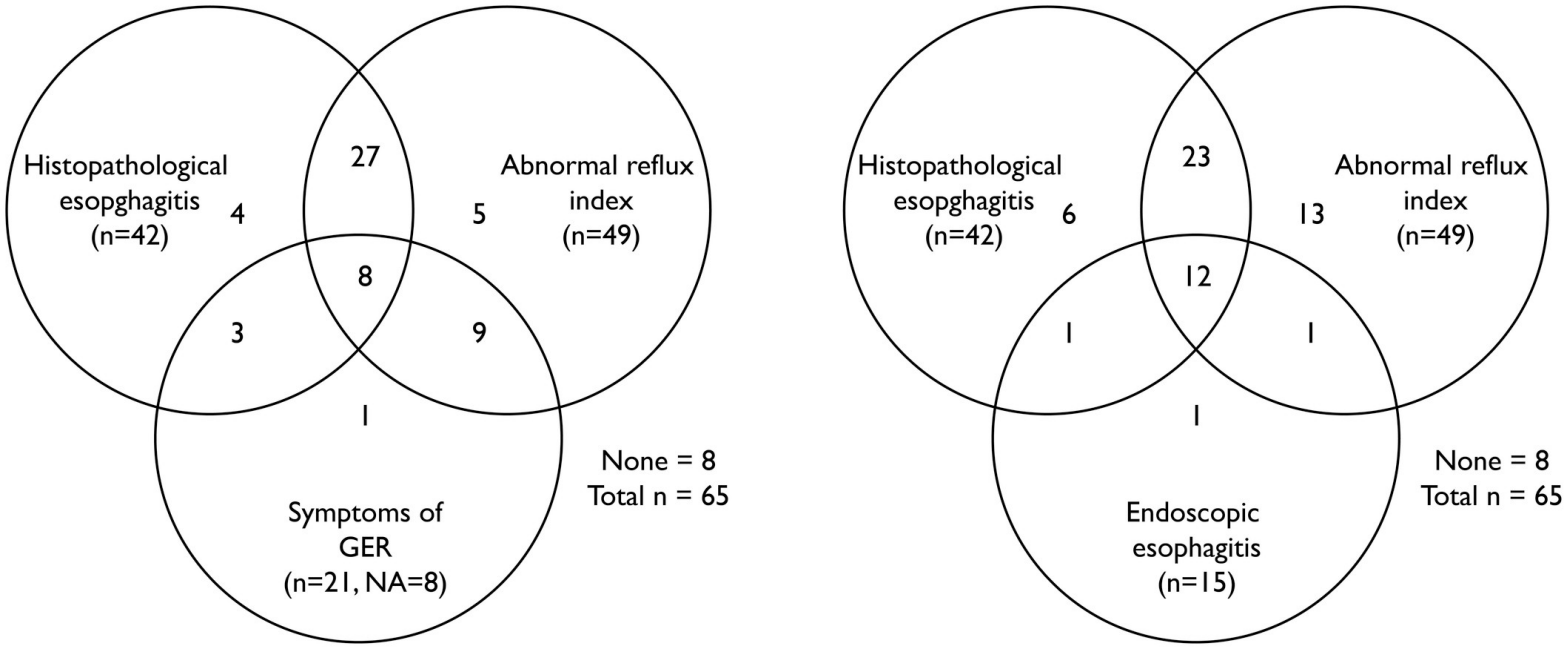
Venn diagram showing the overlap of patients regarding the variables included in logistic regressions. The numbers in each section represent the number of patients that present the particular variable, for overlapping sections the patient presents all the overlapping variables (please note that the size of the section does not correspond to the number of patients presenting the variable/s).

The Venn diagram (Fig 2) illustrates the overlap of patients regarding the variables included in logistic regressions. Patient demographics by histopathological esophagitis are summarised in Table 3 and the results of follow-up procedures in Table 2. Dysphagia was more common in patients with histopathological esophagitis, as was anastomotic stricture, abnormal diet for age, endoscopic esophagitis, hiatal hernia, Boix-Ochoa score and the proportion of patients with abnormal reflux index. The proportion of patients treated with PPI was lower in the esophagitis group (53.6% of patients with esophagitis grade I, 61.5% of patients with grade II, the only patient with grade III and 76.2% of patients without histopathological esophagitis were treated with PPI). The proportion of patients reporting symptoms of GER was also lower in the esophagitis group. Intestinal metaplasia was present in one patient at one year of age.

**Table 3:** Patient demographics by histopathological esophagitis.

Factor	Group	Esophagitis^+^ (n = 42)	No esophagitis (n = 23)
**Background**			
Age (median [IQR])		1.46 [1.18, 3.34]	1.25 [1.05, 14.53]
Sex (%)	Female	20 (47.6)	11 (47.8)
	Male	22 (52.4)	12 (52.2)
Gestational age w (median [IQR])		38.14 [35.07, 39.71]	38.85 [36.85, 40.07]
Birth length Z-score (median [IQR])		-0.06 [-0.66, 0.67] NA = 1	0.48 [-0.29, 0.72]
Birth weight Z-score (median [IQR])		0.51 [-0.96, 0.21]	-0.05 [-0.87, 0.54]
Gross type (%)	A	1 (2.4)	1 (4.3)
	B	1 (2.4)	0 (0.0)
	C	39 (92.9)	20 (87.0)
	D	1 (2.4)	2 (8.7)
	E	0 (0.0)	0 (0.0)
**Operation**			
Type of esophageal correction* (%)	PDA	40 (95.2)	22 (95.7)
	DPA	2 (4.8)	1 (4.3)
Anastomotic stricture (%)		16 (38.1)	6 (26.1)
Nissen fundoplication (%)		2 (4.8)	1 (4.3)
**At follow up**			
Z-score for height (median [IQR])		0.02 [-1.12, 0.67] NA = 18	0.19 [-1.14, 0.96] NA = 11
Z-score for weight (median [IQR])		-0.35 [-1.37, 0.27] NA = 5	0.13 [-1.05, 0.78] NA = 2
Proton pump inhibitor^ (%)		24 (57.1)	16 (76.2) NA = 2
Symptoms of GER (%)		11 (30.6) NA = 6	10 (47.6) NA = 2
Dysphagia (%)		21 (51.2) NA = 1	8 (36.4) NA = 1
Abnormal diet for age (%)		18 (43.9) NA = 1	4 (18.2) NA = 1
Gastrostomy (%)		6 (14.3)	1 (4.3)
Age group (%)	Children	32 (76.2)	15 (65.2)
	Adolescents	10 (23.8)	8 (34.8)

^+^According to Ismail-Beigi [32].

*PDA: primary direct anastomosis; DPA: delayed primary anastomosis.

^At follow-up, all children were treated at least until 1 year of age.

We found no significant associations between histopathological esophagitis and endoscopic esophagitis, symptoms of GER, hiatus hernia or age group. Abnormal reflux index was the only independent predictor of histopathological esophagitis (Table 4). Abnormal reflux index had a sensitivity of 83.3% (95% CI 68.8–93) and a specificity of 39.1% (95% CI 19.7–61.5). However, it has to be added that all patients with histopathological esophagitis that had normal reflux index (n = 7, 16.6% of patients with histopathological esophagitis) had esophagitis grade I.

**Table 4:** Logistic regression model for factors associated with histopathological esophagitis.

*Logistic regressions*	odds ratio	95%CI	p value	n
Abnormal reflux-index	3.21	1–10.3	0.0497	65
Endoscopic esophagitis	4.71	0.959–23.1	0.056	65
Hiatal hernia	1.76	0.48–6.45	0.394	60
Symptoms of GER	0.48	0.159–1.47	0.201	57
Age group (adolescent)	0.59	0.19–1.78	0.347	65
*Multiple logistic regression* *	odds ratio	95% CI	p value	53
Abnormal reflux index	4.32	1.03–18	0.045	
Symptoms of GER	0.27	0.07–1.02	0.053	
Endoscopic esophagitis	5.17	0.87–30.9	0.071	

*Hiatal hernia and age group were excluded from the final model by stepwise selection.

## Discussion

We found that approximately two thirds of the patients had histopathological esophagitis without any significant difference between the two age groups. Abnormal reflux index measured by ambulatory 24h-pH-tests was found to be an independent predictor of histopathological esophagitis. Neither endoscopic esophagitis, symptoms of GER, hiatal hernia nor age group were significant predictors of histopathological esophagitis.

The incidence of histopathological esophagitis is in line with the reports by Schalamon et al. [41] and Petit et al. [29] who reported histopathological esophagitis in 79.7% (n = 59) and 52% of patients (n = 38) at a median age of 15 and 4.9 years, respectively. Moreover, the distribution of histopathological esophagitis from grade I-III was similar to the report by Schalamon et al. [41]. Koivusalo et al. [26] found histopathological esophagitis in 36% of patients (n = 54) at one year of age and 42% (n = 31) at 15 years of age. They reported an overall esophagitis of 52% (n = 109) in different age groups, however, they did not report how many patients were treated with PPI. Pedersen et al. [28] reported histopathological esophagitis in 44.1% of patients (n = 26), with a median age of 10.2 years, 32.2% of the patients were treated with PPI. In summary, the prevalence of histopathological esophagitis was high in our study despite PPI treatment in accordance with recommendations [18], reflecting the complexity of the esophageal morbidity in patients treated for EA and the lack of adequate medication.

Esophageal biopsies are important in monitoring the treatment of patients with EA (statement 5, 6 and 7; ESPGHAN/ NASPGHAN guidelines regarding patients with EA) [18]. They reflect the added effects of different conditions on the esophageal structure and should be considered in the evaluation of the morbidity burden on the esophagus. Moreover, one of the goals of PPI treatment and EA follow-up programmes is to diagnose and prevent histopathological changes in the esophagus (discussion on statement 7, ESPGHAN/ NASPGHAN guidelines regarding patients with EA) [18]. Nevertheless, there are few studies in children/adolescents treated for EA that consider histopathological esophagitis as the outcome and examine pH-metry’s association to it. Castilloux et al. [27] examined this association but only in six patients and reported esophagitis in all patients not treated with PPI. Pedersen et al. [28] reported that 55.2% (n = 32) of patients had an abnormal reflux index. Petit et al. [29] examined risk factors for histopathological esophagitis and found an association to recurrent anastomotic strictures. The present study examined the prediction capabilities of pH-metry in relation to histopathological esophagitis and found a significant predictive value. However, considering that 16% of the patients with histopathological esophagitis (all grade I) were not detected by pH-metry and that both Schalamon et al. [41] and Koivusalo et al. [26] reported progression from esophagitis group 0 and I to group II-III (Schalamon et al. [41] in as many as 25 of 29 patients (86%) within six years after birth); the authors do not recommend the use of pH-metry as a substitute for esophageal biopsies. Possible implications of the results might be that upper endoscopy and esophageal biopsies could be postponed in infants with normal reflux index, similar to the strategy proposed by ESPGHAN/ NASPGHAN guidelines regarding patients with EA who are asymptomatic at discontinuation of PPI [18]. This approach may diminish the episodes of general anaesthesia in early infancy without compromising the patient’s safety since no esophagitis grade II or III were missed.

Endoscopic esophagitis was not a predictor of histopathological esophagitis. The results highlight once again the benefits of biopsies and are in line with Pedersen et al. [28], who found no association between these variables when they studied endoscopic esophagitis as the outcome.

The age group was not a predictor of histopathological esophagitis in our patients with EA. These results are in accordance with Sistonen et al. [42], they reported that 39% of adults treated for EA had histological changes related to esophagitis which were at the same level as in children from the same centre [26]. Koivusalo et al. [26] reported no difference in histopathological esophagitis between age groups.

Symptoms of GER were not independent predictor of histopathological esophagitis. A substantial number of patients without clinical symptoms of GER had histopathological esophagitis (Fig 2). Consequently, symptoms are not reliable as a basis for conducting treatment or monitoring for esophagitis in children treated for EA. Our finding is in agreement with the ESPGHAN/ NASPGHAN guidelines [18] (statement 5, 6 and 7). This is in contrast to the conclusions reached by Koivusalo et al. [26], but can be supported by others showing no association between symptoms of GER and abnormal reflux index [2, 27].

Hiatal hernia had no association with histopathological esophagitis. Many studies examine the association of hiatal hernia with GER and endoscopic esophagitis but, to our knowledge, no reports on the association with histopathological esophagitis have been published.

The proportion of patients treated with PPI at follow-up was lower in the group with histopathological esophagitis. This supports the view that GER is causative of esophagitis [43]. On the other hand, all children were treated with PPI for one year and still 68% had histopathological esophagitis and 72.3% had abnormal reflux index. In the adolescent study group only 1/3 of the patients were treated with PPI but the proportion of patients with histopathological esophagitis was similar or lower than the children’s group and more than half of the group had abnormal reflux index. This raises questions about the treatment compliance, the effectiveness of PPI treatment, as well as the complexity of the esophageal morbidity with non-acidic reflux, bolus transit time, etc. Our group, among others, has previously investigated the effectiveness of PPI treatment and found no effect on stricture formation [44–46].

The strengths of this study were the inclusion of a fairly large study population with well-defined age groups of children and adolescents after repair of EA in a pre-established ongoing follow-up programme. Moreover, the current study is, to our knowledge, besides a report on six patients, the only study to report the association between pH-metry and histopathologic esophagitis in children and adolescents with EA. Our study population is representative of a general population of patients treated for EA [47].

The limitations of the study are its observational design, the loss of some patients with incomplete data, the exclusion of the impedance data in the analyses and the limited number of predictors included based on the sample size. The confidence intervals in the logistic regressions of endoscopic esophagitis and abnormal reflux index are wide, reflecting an uncertainty in the effect of the predictor due to spread and sample size.

## Conclusion

We found that the prevalence of histopathological esophagitis was high despite PPI treatment in accordance with recommendations. No significant difference between the two age groups was seen. Abnormal reflux index measured by ambulatory 24h-pH-tests was found to be an independent predictor of histopathological esophagitis. However, we cannot recommend the use of pH-metry as a substitute for esophageal biopsies and future studies are needed to elucidate if esophageal biopsies might be postponed in infants with normal reflux index.
